# Supplementary material for: Synthesis and Characterization of Chelating Hyperbranched Polyester Nanoparticles for Cd(II) Ion Removal from Water
Source: Molecules. 2022 Jun 7;27(12):3656. doi: 10.3390/molecules27123656 (PMC9231149; doi:10.3390/molecules27123656)
Supplement: Supplementary file 1 [file molecules-27-03656-s001.zip › molecules-1766738-supplementary.pdf]

molecules

Supplementary Data

Synthesis and Characterization of Chelating Hyperbranched Polyester Nanoparticles for Cd(II) Ion Removal from Water

Faten Alregeb [1,2], Fawwaz Khalili [2], Bassam Sweileh [2] and Dalia Khalil Ali [3,*]

1 Faculty of Science Arts and Sciences, Middle East University, Amman 11610, Jordan; fregab@meu.edu.jo
2 Department of Chemistry, Faculty of Science, The University of Jordan, Amman 1118, Jordan; fkhalili@ju.edu.jo (F.K.); sweilehb@ju.edu.jo (B.S.)
3 Faculty of Allied Medical Sciences, Isra University, Amman 11622, Jordan; dalia.ali@iu.edu.jo (D.A.)
* Correspondence: dalia.ali@iu.edu.jo; Tel.: +962-79727417

Citation: Alregeb, F.; Khalili, F.; Sweileh, B.; Ali, D. Synthesis and Characterization of Chelating Hyperbranched Polyester Nanoparticles for Cd(II) Ion Removal from Water. *Molecules* **2022**, *27*, 3656. https://doi.org/10.3390/molecules27123656

Academic Editors: Shaojun Yuan, Ying Liang, Changkun Liu and Xiaoying Liu

Received: 26 May 2022
Accepted: 4 June 2022
Published: 7 June 2022

Publisher's Note: MDPI stays neutral with regard to jurisdictional claims in published maps and institutional affiliations.

Copyright: © 2022 by the authors. Submitted for possible open access publication under the terms and conditions of the Creative Commons Attribution (CC BY) license (https://creativecommons.org/licenses/by/4.0/).

Table S1. Cd(II) uptake by the polyester as a function of contact time, at pH=4.00, Temp 25 °C, 35 °C 45 °C and initial concentration of 150 ppm.

pH=4.00 and 25.0 °C				
Time (h)	C_e (mg/L)	Uptake(mg/g)	% Uptake	t/q_t (hour g/mg)
0.5	139.03	2.68	7.31	0.19
1	120.32	7.42	19.79	0.13
2	113.80	9.05	24.13	0.22
4	110.52	9.87	26.32	0.41
6	109.22	10.20	27.19	0.59
8	102.02	12.00	31.99	0.67
10	98.33	12.92	34.45	0.77
18	90.12	14.97	39.92	1.20
24	83.90	16.53	44.07	1.45
48	38.30	27.93	74.47	1.72
pH=4.00 and 35.0°C				
0.5	139.80	3.06	6.80	0.16
1	126.30	7.11	15.80	0.14
2	96.54	16.04	35.64	0.12
4	90.17	17.95	39.89	0.22
6	88.77	18.37	40.82	0.33
8	86.69	18.99	42.46	0.42
10	81.47	20.56	45.69	0.49
18	60.31	26.91	59.79	0.67
24	27.03	36.89	81.98	0.65
48	24.50	37.65	83.67	1.27
pH=4.00 and 45.0°C				
0.5	101.00	14.70	32.67	0.034
1	100.50	14.85	33.00	0.067
2	98.40	15.48	34.4	0.13
4	94.85	16.55	36.77	0.24

6	90.31	17.91	39.79	0.34
8	80.62	20.81	46.25	0.38
10	79.42	21.17	47.05	0.47
18	59.22	27.23	60.52	0.66
24	19.56	39.13	86.96	0.61
48	20.32	38.90	86.45	1.23
